# Use of ^18^F-fludeoxyglucose positron emission tomography-CT in the management of breast implant-associated anaplastic large cell lymphoma

**DOI:** 10.1259/bjrcr.20150424

**Published:** 2016-07-28

**Authors:** Sapna Ladani, Kalliope Valassiadou, Yvette Griffin, Fiona Miall

**Affiliations:** ^1^Department of Haematology, University Hospitals of Leicester NHS Trust, Leicester, UK; ^2^Department of Breast Surgery, University Hospitals of Leicester NHS Trust, Leicester, UK; ^3^Department of Radiology, University Hospitals of Leicester NHS Trust, Leicester, UK

## Abstract

The prognosis and preferred management of breast implant-associated anaplastic large cell lymphoma is dependent on whether lymphoproliferative cells are confined to within the fibrous capsule, in an effusion or lining the fibrous capsule, or if there is spread beyond the capsule in the form of a mass lesion. We describe a case where ^18^F-fludeoxyglucose positron emission tomography-CT was used to confirm localized disease and guide management decisions.

## Summary

Breast implant-associated anaplastic large cell lymphoma (BIA-ALCL) is an extremely rare but increasingly recognized subtype of T-cell lymphoma. ALCL in a patient with a breast implant was first reported in 1997.^1^ Publication of further cases and a case–control study by de Jong et al^2^ led to the US Food and Drug Administration identifying an association between breast implants and ALCL in 2011.^3^ A recent review updated the published series to 173 cases worldwide^4^ and current estimates suggest that there have been up to 250 cases worldwide.^5^

The definitive cause and pathogenesis have still not been identified.^6^ BIA-ALCL was initially treated as other subtypes of T-cell lymphoma with chemotherapy. However, it is increasingly thought to represent a new subtype of ALCL with an indolent course. Current guidelines on how this condition should be managed are based on observational studies, as the rarity of the condition precludes any interventional studies. There is very little in the published literature on the use of imaging, particularly positron emission tomography (PET)-CT scan, in BIA-ALCL. The largest review so far included imaging studies of 44 patients dating between 1997 and 2013 and discusses sensitivity and specificity for ultrasound, MR, mammography, CT and PET-CT scan.^7^

We describe a case of a 66-year-old female presenting with BIA-ALCL where ^18^F-fludeoxyglucose (FDG) PET-CT scan was used to guide treatment decisions. We also review some of the current literature that we used to aid management decisions.

## Case report

This 66-year-old female was diagnosed with symptomatic carcinoma of the right breast in August 2010. She went on to have a wide local excision and sentinel node biopsy, which was followed by skin-sparing mastectomy and insertion of tissue expander owing to close margins. Histological examination showed a T2N0M0 invasive ductal carcinoma, which was oestrogen receptor positive and HER-2 negative, with no lymphovascular invasion. Her post-operative course was unremarkable and she was started on adjuvant treatment with anastrozole 1 mg daily. In February 2011, she had an exchange of the tissue expander for a permanent fixed-volume textured anatomical cohesive silicone gel implant. This was followed by nipple reconstruction under local anaesthetic in October 2011.

**Figure 1.**
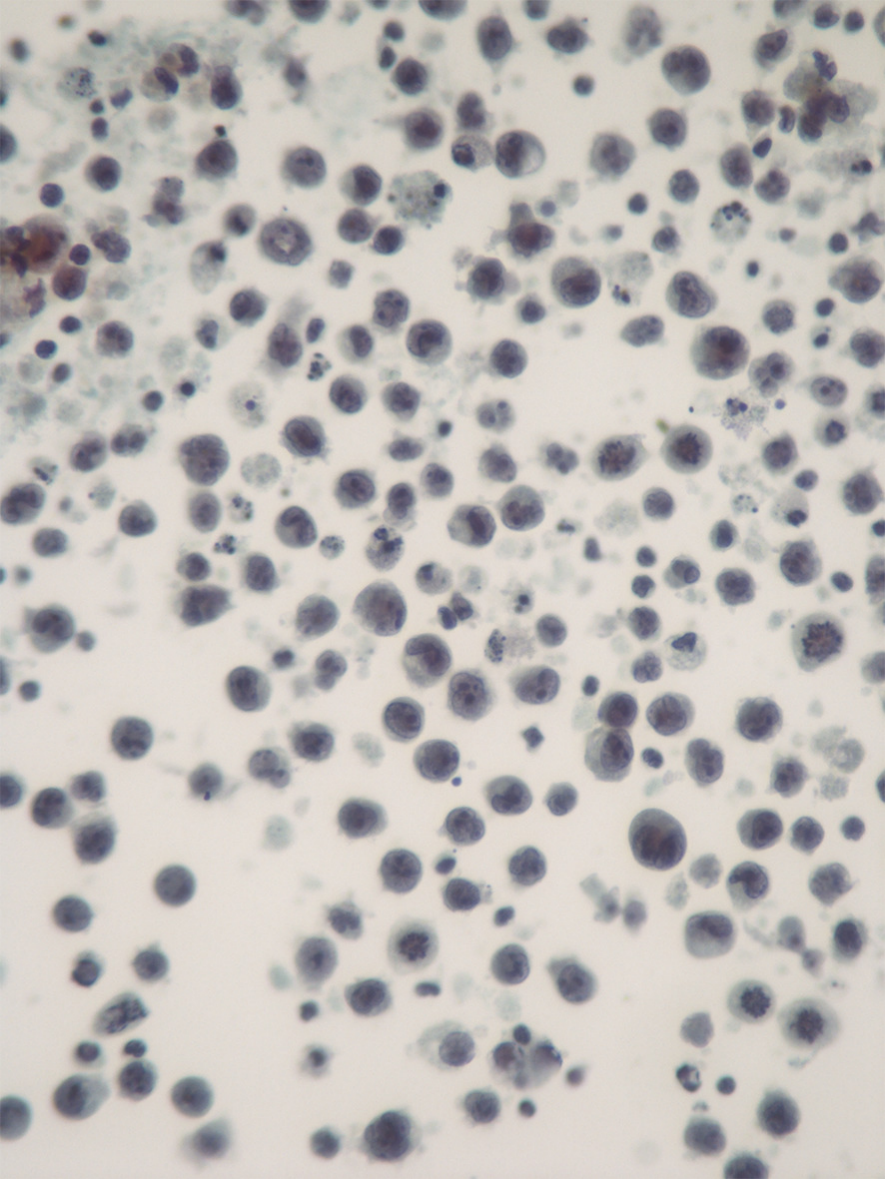
Pleomorphic lymphoid cells present in the aspirated effusion. Subsequent immunohistochemical staining confirmed this to be breast implant-associated anaplastic large cell lymphoma.

**Figure 2.**
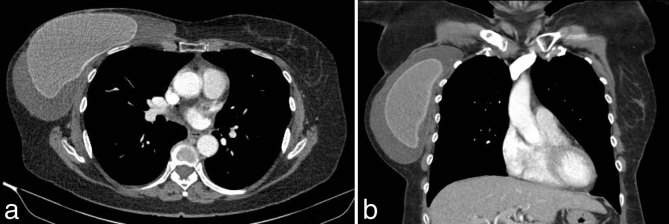
Pre-operative contrast-enhanced CT scan (a) axial and (b) coronal views demonstrating right mastectomy with implant and associated effusion in the implant cavity.

In June 2014, she developed pruritus over the right reconstructed breast and within 3 weeks re-presented with a very enlarged right breast. An ultrasound scan confirmed the presence of a new large seroma, and 600 ml of straw-coloured fluid was aspirated and sent for cytology and microbiology. Following aspiration, it was clinically evident that the implant looked intact, as there was no alteration in its shape. Cytological examination revealed malignancy, showing a population of lymphoid cells (Figure 1) that were positive for CD45, CD30, CD3, CD2 and CD4, and negative for EMA, CD20, CD79a and ALK-1. T-cell receptor gene rearrangement studies confirmed a monoclonal population of T-cells and the diagnosis of BIA-ALCL was established. A contrast-enhanced CT scan of the chest, abdomen and pelvis confirmed an effusion within the right breast implant cavity (Figure 2) and showed no other evidence of disease.

The patient went on to have removal of the implant and complete capsulectomy. There was no evidence of macroscopic rupture of the implant. Histological examination showed focal aggregates of malignant lymphoid cells within the fibrin (capsule) lining the implant cavity. No infiltration outside the cavity was seen. The case was reported to the Medicines and Healthcare Products Regulatory Agency, UK.

**Figure 3.**
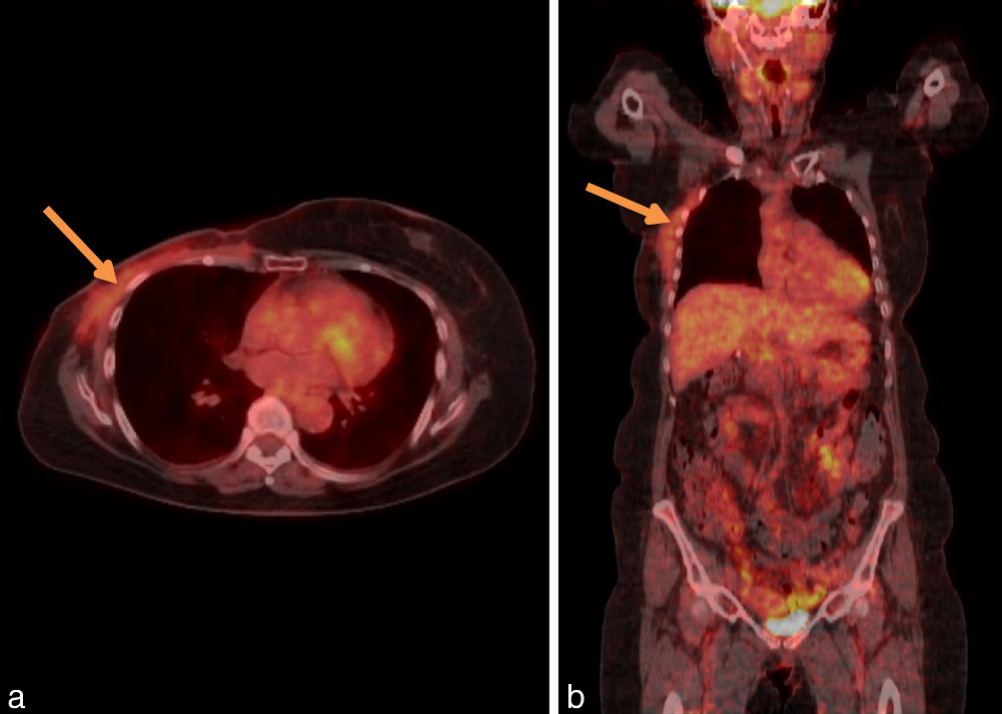
(a) Axial and (b) coronal views ofpost-operative ^18^F-fludeoxyglucose positron emission tomography-CT demonstrating low level metabolic activity at the operative site, likely owingtopost-operative inflammation.

**Figure 4.**
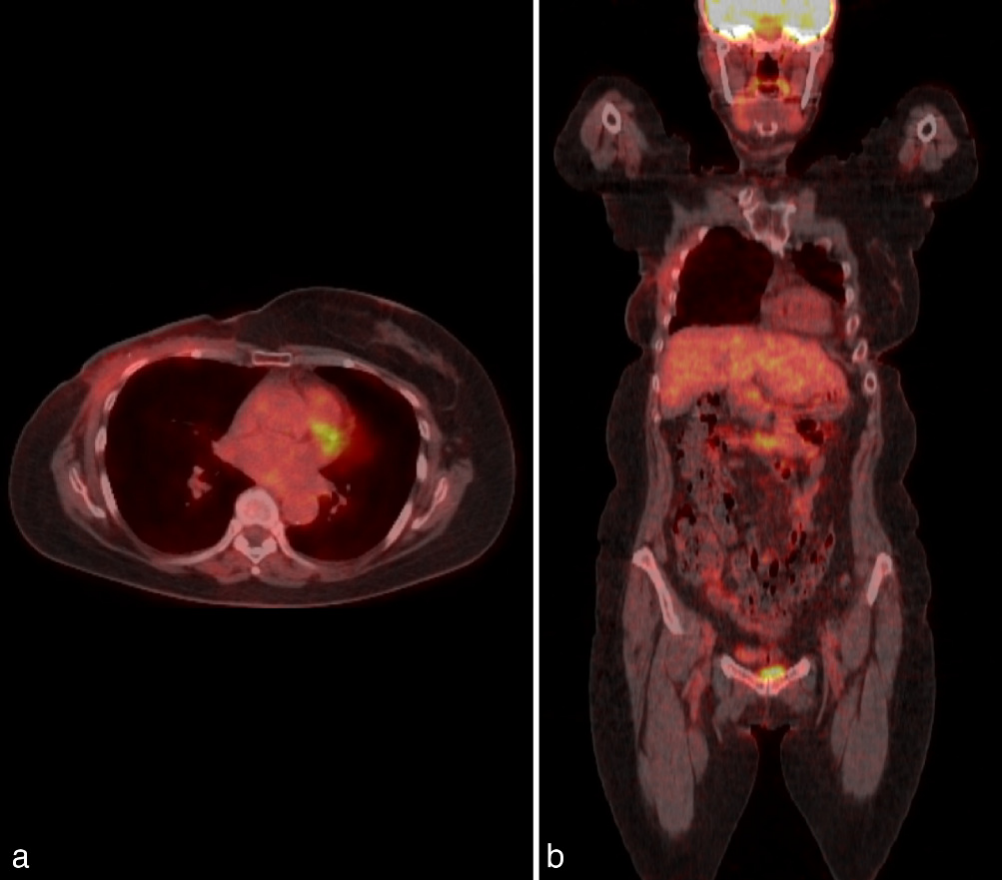
(a) Axial and (b) coronal views of ^18^F-fludeoxyglucose positron emission tomography-CT performed 3 months postoperatively demonstrating reduction of previous 18F-fludeoxyglucose uptake.

**Figure 5.**
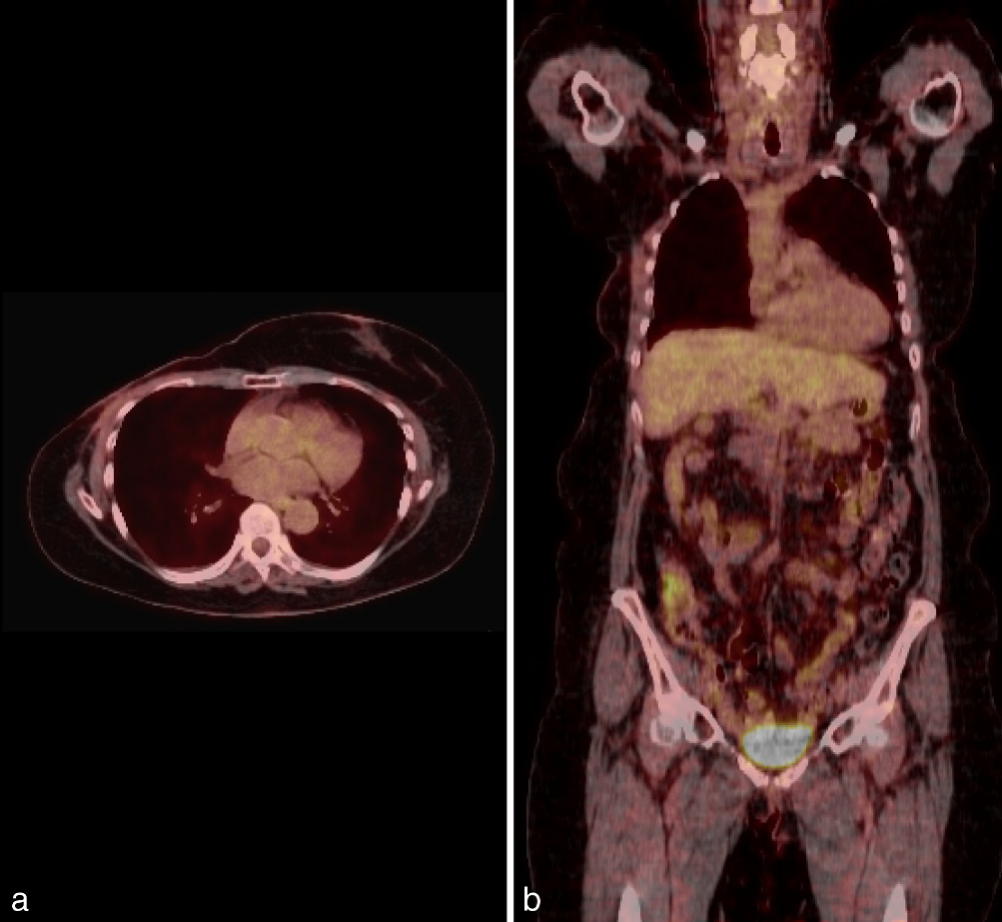
(a) Axial and (b) coronal views of 18F-fludeoxyglucose PET-CT scan performed 12 months postoperatively showing complete resolution of previous ^18^F-fludeoxyglucose uptake with no evidence of ^18^F-fludeoxyglucose-avid disease.

A post-operative FDG PET-CT scan showed only low level metabolic activity [maximum standardized uptake (SUV) value of 2.4] that was limited to the chest wall at the recent operative site, which was considered postsurgical (Figure 3). Based on the clinicopathological assessment, review of the emerging medical literature and imaging results of CT and FDG PET-CT scan confirming no mass lesions, a decision was made to watch and wait. At 3 months, there was no evidence of recurrence on repeat FDG PET-CT scan (Figure 4). There was again only low level FDG uptake with a maximum SUV of 2.1 and no reaccumulation of the seroma. A 12-month FDG PET-CT scan (Figure 5) showed resolution of the previous FDG uptake, with no evidence of FDG-avid disease.

## Discussion

Nodal anaplastic lymphoma kinase-negative ALCL generally has a guarded prognosis with 40% 5-year survival. However, it is becoming increasingly clear that BIA-ALCL represents a separate disease entity.

Chronic inflammation caused by the presence of an implant, particularly with textured implants, and possibly also by contamination with bacterial fragments has been postulated to have a role in the pathogenesis of BIA-ALCL, although further evidence is needed to support causality. Chronic inflammation is thought to lead to antigenic stimulation of toll-like receptors on immune cells. It has been postulated that this acts as a trigger for the development of BIA-ALCL.^8^ This reactive entity has an indolent course with good prognosis. However, in a minority of cases, constant antigenic stimulation leads to the accumulation of multiple oncogenic mutations in T cells, leading to development of dominant clones. These clones acquire some features that are common in more aggressive systemic CD30+ lymphomas such as the ability to invade tissues and metastasize. These patients are likely to suffer from a more aggressive disease with a worse prognosis. Currently, it is not possible to identify a type of implant (silicone *vs* saline) or a reason for implant (reconstruction *vs* aesthetic augmentation) associated with a smaller or greater risk.^3^

Miranda et al^9^ obtained long-term follow-up data for 60 patients with BIA-ALCL. 42 of these patients presented with an effusion in the implant cavity, whereas 18 presented with a mass. Following surgery, 71% had chemotherapy, 7% radiotherapy and 22% followed a watch and wait approach. 93% of patients with effusion achieved complete remission, with one patient dying from unrelated causes; 72% of patients with a mass achieved complete remission; 17% died as a result of their disease. This suggests that implant-associated ALCL is not a completely benign entity and risk stratification is needed. The authors suggest that patients without a mass can be treated more conservatively, with perhaps removal of the implant with capsulectomy alone. They also suggest that those with a mass may have a more aggressive course, so may need removal of the implant and systemic therapy, which still needs to be defined. National Comprehensive Cancer Network treatment guidelines now suggest that a watch and wait approach following surgery in those without a mass may be considered.^10^

There is increasing emphasis on distinguishing those cases presenting with a solitary effusion who are likely to have an indolent course from those with mass lesions who are likely to have more aggressive disease. A recent consensus paper suggested an algorithm for work-up that includes FDG PET-CT.^11^ Aggressive T-cell lymphomas, including anaplastic lymphoma kinase-ALCL, have been found to be FDG PET-CT avid.^12,13^ Adrada et al^7^ found that FDG PET-CT had better sensitivity in detecting mass lesions in BIA-ALCL compared with ultrasound, CT and MRI (64 *vs* 46, 50 and 50%, respectively). Expected SUV values for cases of BIA-ALCL with an effusion or mass lesion have yet to be established.

In this case, as in others, we have used a staging FDG PET-CT in addition to CT scan, intraoperative findings and histological analysis to confirm the absence of a mass. This contributed to the decision to withhold chemotherapy and monitor the patient. We went on to repeat the FDG PET-CT scan to confirm maintained response and propose this as a radiological surveillance strategy in this subtype of lymphoma, which until recently was being treated with systemic chemotherapy. Maintained negativity by FDG PET-CT scan further reassured the patient and her treating clinicians of their watch and wait approach.

## Conclusions

In this rare group of patients, evidence is limited and long-term follow-up data is needed. We have used FDG PET-CT scan at staging to confirm isolated disease in a patient with BIA-ALCL to guide management and for radiological surveillance.

## Learning points

1. Patients with BIA-ALCL presenting with a solitary effusion need to be distinguished from those with mass lesions.
2. Given the rarity of the condition, optimal management is still to be established. However, a conservative approach following removal of implant and capsulectomy could be considered for some patients with no evidence of mass lesion, without the use of chemotherapy. Use of chemotherapy in patients presenting with a mass is yet to be defined.
3. FDG PET-CT scan has been shown to detect mass lesions in patients with BIA-ALCL.

## Consent

Written informed consent was obtained from the patient for publication of this case report, including accompanying images.
